# Potent anti-inflammatory responses: Role of hydrogen in IL-1α dominated early phase systemic inflammation

**DOI:** 10.3389/fphar.2023.1138762

**Published:** 2023-03-17

**Authors:** Youzhen Wei, Kun Wang, Yafang Zhang, Yi Duan, Yan Tian, Hongling Yin, Xuelian Fu, Zuan Ma, Jianjun Zhou, Min Yu, Qingbin Ni, Wenjie Tang

**Affiliations:** ^1^ Research Institute of Heart Failure, Research Center for Translational Medicine & Key Laboratory of Arrhythmias of the Ministry of Education of China, East Hospital, Tongji University School of Medicine, Shanghai, China; ^2^ Hydrogen Medicine Center, The Affiliated Taian City Central Hospital of Qingdao University, Taian, Shandong, China; ^3^ Research Center for Translational Medicine, Jinan People’s Hospital, Shandong First Medical University, Jinan, Shandong, China; ^4^ Office of Academic Research, Taishan Vocational College of Nursing, Taian, Shandong, China; ^5^ Department of Neonatology and NICU, The Affiliated Taian City Central Hospital of Qingdao University, Taian, Shandong, China; ^6^ The Key Laboratory of Metabolism and Molecular Medicine, The Ministry of Education, Department of Biochemistry and Molecular Biology, School of Basic Medicine, Fudan University, Shanghai, China; ^7^ Research Institute of Regenerative Medicine, East Hospital, Tongji University, Shanghai, China

**Keywords:** hydrogen, HIF-1α, IL-1α, chemokine, inflammation

## Abstract

**Introduction:** It has been proven that hydrogen has obvious anti-inflammatory effects in animal experiments and clinical practice. However, the early dynamic process of the inflammatory response caused by lipopolysaccharide (LPS) and the anti-inflammatory effect of hydrogen has not been definitively reported.

**Methods:** Inflammation in male C57/BL6J mice or RAW264.7 cells was induced with LPS, for which hydrogen was immediately administered until samples were taken. Pathological changes in lung tissue were assessed using hematoxylin and eosin (HE) staining. Levels of inflammatory factors in serum were determined using liquid protein chip. The mRNA levels of chemotactic factors in lung tissues, leukocytes, and peritoneal macrophages were measured by qRT-PCR. The expression levels of IL-1α and HIF-1α were measured by immunocytochemistry.

**Results:** Hydrogen alleviated LPS-induced inflammatory infiltration in the lung tissues of mice. Among the 23 inflammatory factors screened, LPS-induced upregulation of IL-1α etc. was significantly inhibited by hydrogen within 1 hour. The mRNA expression of MCP-1, MIP-1α, G-CSF, and RANTES was inhibited obviously by hydrogen at 0.5 and 1 h in mouse peritoneal macrophages. In addition, hydrogen significantly blocked LPS or H2O2-induced upregulation of HIF-1α, and IL-1α in 0.5 h in RAW264.7 cells.

**Discussion:** The results suggested that hydrogen is potentially inhibitive against inflammation by inhibiting HIF-1α and IL-1α release at early occurrence. The target of the inhibitive LPS-induced-inflammatory action of hydrogen is chemokines in macrophages in the peritoneal cavity. This study provides direct experimental evidence for quickly controlling inflammation with the translational application of a hydrogen-assisted protocol.

## Introduction

Inflammation represents an adaptive response of the body to external stimuli, which is a very common but complex process with dual effects of defense and injury (Cavaillon, 2018). Although the pathogeneses of inflammation-associated diseases (such as pneumonia, pancreatitis, and hepatitis) are different, the course of the disease is often accompanied by inflammatory cell infiltration, inflammatory mediator release, and the production of a variety of cytokines, free oxygen radicals, and other active substances (Miller et al., 2018). These active substances, produced in large quantities, can directly damage the integrity of blood vessels and surrounding tissues, leading to functional abnormalities. Therefore, anti-inflammation is an important and straightforward strategy for the treatment of these diseases (Liao et al., 2019).

Hydrogen is a colorless, odorless, tasteless gas that has been proven to have obvious anti-inflammatory effects in animal experiments (Li et al., 2019; Li et al., 2021; Mu et al., 2022; Yin et al., 2022) and clinical practice (Guan et al., 2020; Wang et al., 2020; Yang et al., 2020; Singh et al., 2021; Botek et al., 2022; Luo et al., 2022). However, the early dynamic process of the inflammatory response caused by lipopolysaccharide (LPS) and the anti-inflammatory effect of hydrogen has not been definitively reported. Our previous study found that hydrogen intervention in a LPS-induced acute systemic inflammation mouse model significantly reduced inflammatory damage, inhibited LPS-induced serum lactate increase, improved survival rate, and prolonged survival time. The time points were selected at 6, 12, 24, and 48 h to detect and analyze plasma inflammatory factors. The results showed that the anti-inflammatory effect of hydrogen occurred much earlier than expected, and the secretion of inflammatory factors changed distinctly at approximately 6 h (Yin et al., 2022).

Therefore, this study further aimed to ascertain the early dynamic process of the inflammatory response caused by LPS and the anti-inflammatory effect of hydrogen. We selected earlier time points and conducted the following experimental studies, expecting to deliver a scientific and normalized support for the development of optimal clinical treatment plans.

## Materials and methods

### Animal model of the acute systemic inflammatory response

Male C57/BL6J mice (SPF, 22–25 g, 6–8 weeks) were purchased from Shanghai Jihui Experimental Animal Feeding Co., Ltd., and kept under standard conditions of illumination with 12 h light/dark cycles at 25°C ± 2°C with free access to food and tap water. All experimental procedures were carried out in strict accordance with the principles of laboratory animal care, approved by the Animal Ethics Committee of Tongji University.

### Animal experimental design, grouping, and hydrogen chamber assembly

To study the anti-inflammatory effect of hydrogen inhalation in the animal model of acute systemic inflammatory response, mice were randomly divided into a normal control group, an LPS-induced acute systemic inflammatory model group, and a hydrogen administration group. LPS (Sigma-Aldrich; St Louis, MO, United States) freshly prepared with normal saline at a concentration of 2 mg/mL was injected into the peritoneal cavity of the mice at 2 mg/kg based on the body weight of mice. After creation of the inflammation model, mice were immediately placed in a hydrogen treatment warehouse (Supplementary Figure S1A) with 42% hydrogen and 21% oxygen for continuous hydrogen absorption until the samples were taken. The warehouse body was transparent so that the mice could be observed.

The hydrogen supply device consisted of four parts: hydrogen-oxygen atomizer, nitrogen inlet device, oxygen controller and flow filter chamber (Supplementary Figures S1B–E). The atomizer was a Hydrogen and Oxygen Haze Machine (AMS-H-03) provided by Shanghai Asclepius MediTec Co., Ltd., with gas production set at 3,000 mL/min, H_2_:O_2_ = 2:1. The gas generated was imported into the tank through a closed silica-gel pipe. The nitrogen inlet device was purchased from Shanghai Shencai Gas Technology Co., Ltd., and nitrogen (endotoxin free, purity > 99.99%) was introduced into the tank to reduce the O_2_ concentration to 21%. The gas flow filter chamber, which contained a silica-gel desiccant and sodium lime (YZB/Shanghai 2136-54A-2010, Shanghai Nahui Drying Reagent Factory), controlled the moisture and CO_2_ released by the animals.

### Specimen collection

After isoflurane anesthesia, blood (approximately 0.5 mL) was immediately collected from the eye socket of each mouse in different groups. For the cytokine and chemokine detection, blood samples were collected in 28 mice at 1, 3, and 6 h time points. After blood autocoagulation for 1 h, serum was extracted by centrifugation at 4°C and 4,000 rpm for 5 min. For the gene expression level test, blood samples were collected from other 15 mice at 0.5, and 1 h time points in heparin tubes. After centrifugation at 4°C and 3,000 rpm for 10 min, blood erythrocytes were lysed using Red Blood Cell Lysis Buffer (Beyotime Biotechnology, Jiangsu, China). After lysis, the samples were centrifuged immediately at 3,000 rpm for 5 min at room temperature, and the supernatant was decanted. After washing with phosphate-buffered saline (PBS) twice, blood leukocytes were collected, lysed with TRIzol Reagent (Thermo Fisher Scientific, United States) and preserved at −80°C.

For the histological examinations, two lungs from each mouse treated with LPS and/or hydrogen for 3 h were removed after rapid thoracotomy. One lung was placed in liquid nitrogen and stored at −80°C, and the other lung was placed in 10% neutral buffered formalin for 24 h at room temperature. Peritoneal macrophages were collected from each mouse using a micro-syringe and cold 6 mL PBS. Cell pellets were obtained after centrifugation at 4°C and 3,000 rpm for 5 min.

### Histological examinations

Fixed lung tissues were embedded in paraffin. All specimens were cut into 5 μm thick sections and stained with hematoxylin and eosin (HE). Samples were examined immediately by Leica DM Microscopes (DM 2500B, Leica, Wetzlar, Germany) and photographed at ×20 or ×40 magnification.

### Cytokine and chemokine detection

The serum levels of 23 cytokines and chemokines (IL-1α, IL-1β, IL-2, IL-3, IL-4, IL-5, IL-6, IL-9, IL-10, IL-12p40, IL-12p70, IL-13, IL-17, Eotaxin-1 (CCL11), G-CSF (CSF3), GM-CSF (CSF2), IFN-γ, keratinocyte-derived cytokine (KC), MCP-1 (CCL2), MIP-1α (CCL3), MIP-1β (CCL4), RANTES (CCL5), and TNF-α) were assayed using a 23-Plex protein chip (Bio-Plex 200 system, Bio-Rad, Hercules, CA, United States) at 1, 3, and 6 h. Each sample was analyzed in duplicates, and both high- and low-concentration quality controls were used. The actual concentration of each factor in the serum was calculated according to standard curve fitting and dilution ratios.

### Quantitative reverse transcription PCR (qRT-PCR)

The primers and fluorogenic probe were designed and synthesized according to the specific sequences of MCP-1, MIP-1α, G-CSF, RANTES, and Eotaxin-1 genes. Glyceraldehyde-3-phosphate dehydrogenase (GAPDH) was used as the housekeeping gene for normalization of gene expression levels. The sequences of the gene-specific primers used for qRT-PCR analysis are listed in Table 1. Total RNA was respectively extracted from snap-frozen lung tissues, blood leukocytes, and peritoneal macrophages, according to the TRIzol method, and was reverse-transcribed using Thermo Scientific RevertAid cDNA Synthesis Kit (Thermo Fisher Scientific, United States) to produce cDNA. Quantitative real-time PCR was performed with the ABI Prism 7,900 real-time PCR Cycler (Applied Biosystems, Foster City, CA, United States) using SYBR Green UDG Master Mix (Invitrogen, Darmstadt, Germany). PCR conditions were set in step accordance with 95°C for 10 min, 40 cycles of 95°C for 10 s, and 60°C for 30 s. Each sample was conducted in triplicates, and the gene expression levels were calculated relative to the amount of GAPDH using the 2^−ΔΔCT^ method.

**TABLE 1:** Sequences of gene-specific primers used for qRT-PCR analysis.

Gene	Primer sequence 5′to 3′
MCP-1/CCL2-qF	GGT​CCC​TGT​CAT​GCT​TCT​GG
MCP-1/CCL2-qR	CCT​GCT​GCT​GGT​GAT​CCT​CT
RANTES/CCL5-qF	GCC​CTC​ACC​ATC​ATC​CTC​ACT
RANTES/CCL5-qR	GGA​GAG​GTA​GGC​AAA​GCA​GCA
G-CSF/CSF3-qF	GCA​GGC​TCT​ATC​GGG​TAT​TTC​C
G-CSF/CSF3-qR	GCA​GAA​GTG​AAG​GCT​GGC​AT
Eotaxin-1/CCL11-qF	GCT​CAC​GGT​CAC​TTC​CTT​CAC
Eotaxin-1/CCL11-qR	TCT​CTT​TGC​CCA​ACC​TGG​TCT
MIP-1α/CCL3-qF	CTG​CCT​GCT​GCT​TCT​CCT​ACA
MIP-1α/CCL3-qR	GGC​ATT​CAG​TTC​CAG​GTC​AGT​G
GAPDH-F1	TGA​AGG​TCG​GTG​TGA​ACG​GAT​T
GAPDH-R1	CGT​GAG​TGG​AGT​CAT​ACT​GGA​ACA

### Cell culture and treatment

The mouse macrophage cell line (Raw264.7) was purchased from the American Type Culture Collection (ATCC). Cells were seeded at a confluence of approximately 10% in corresponding wells and grown to a confluence of approximately 80% in high-glucose Dulbecco’s modified Eagle’s medium (DMEM) containing 10% fetal bovine serum (FBS) and 1% penicillin and streptomycin (Thermo Fisher Scientific, United States). After that, the cells cultured on glass coverslips were treated with different reagents for 0.5 h, respectively. Three independent experiments were carried out.

The experiment for the effects of hydrogen on LPS-induced inflammation included four groups: Control group, H_2_ group, LPS group, and LPS + H_2_ group. The Control group was cultured with normal DMEM medium and stimulated with PBS in humidified atmosphere containing 5% CO_2_/95% air at 37°C; for 0.5 h the H_2_ group was cultured with normal DMEM medium in humidified atmosphere containing hydrogen mixed gas (60% H_2_, 21% O_2_, 5% CO_2_, and 14% N_2_ (endotoxin free, purity > 99.9%) in air, at 37°C; for 0.5 h with H_2_ produced by hydrogen–oxygen atomizer; the LPS group was cultured with normal DMEM medium and stimulated with LPS (1 μg/mL) in humidified atmosphere containing 5% CO_2_/95% air at 37°C; for 0.5 h the LPS + H_2_ group was cultured with normal DMEM medium and stimulated with LPS in humidified atmosphere containing hydrogen mixed gas in air at 37°C; for 0.5 h.

The experiment for the effects of hydrogen on the H_2_O_2_ (31.26 µM)- challenged inflammatory action included four groups: Control group, H_2_O_2_ group, H_2_O_2_+H_2_ group, and H_2_O_2_+H_2_+H_2_O_2_ group. The Control group was cultured with normal DMEM medium and stimulated with PBS in humidified atmosphere containing 5%CO2/95% air at 37°C for 0.5 h; the H_2_O_2_ group was cultured with normal DMEM medium and stimulated with H_2_O_2_ (Sigma-Aldrich; St Louis, MO, USA) in humidified atmosphere containing 5%CO2/95% air at 37°C for 0.5 h; the H_2_O_2_+H_2_ group was cultured with normal DMEM medium + 60% H_2_ and stimulated with H_2_O_2_ in humidified atmosphere containing hydrogen mixed gas in air at 37°C; for 0.5 h; the H_2_O_2_+H_2_+H_2_O_2_ group was cultured with normal DMEM medium and stimulated with H_2_O_2_ in humidified atmosphere containing hydrogen mixed gas in air for at 37°C; 0.5 h, then with H_2_O_2_ without 60% H_2_ for another 0.5 h.

### Immunocytochemical staining

RAW 264.7 cells after different treatments were fixed with 4% paraformaldehyde (PFA) in phosphate buffer (PB; pH 7.4) for 10 min at room temperature. After fixation, the cultures were washed with PBS and incubated for 1 h with blocking solution (5% bovine serum albumin (BSA) serum (Sigma-Aldrich; St Louis, MO, United States), 0.05% Tween in PBS), and then incubated overnight at 4°C with monoclonal anti-HIF-1α (1:500, Abcam ab179483), FITC-conjugated Affinipure Goat Anti-Rabbit IgG (H + L) (1:100, Proteintech SA00003-2), anti-IL-1α (1:200, Proteintech 16765-1-AP Rabbit), and Cy3-conjugated AffiniPure Goat Anti-Rabbit IgG (H + L) (1:200, Proteintech SA00009-2). Nuclei were counterstained with 4’,6-diamidino-2-phenylindole (DAPI). Cells in each well were examined under a Leica microscope (DMI6000B, Leica, Wetzlar, Germany), and images were captured using a digital video camera (0.55 S 2B). At least six fields from each cell sample in triplicates were acquired. Quantification of the fluorescence intensity was performed using the Photoshop CC software (Adobe Inc., United States).

### Statistical analysis

SPSS 16.0 statistical software (IBM Corp., Armonk, NY, United States) was used for statistical analysis, with data expressed as mean ± standard error of mean (SEM). Significant differences among groups were assessed using one-way analysis of variance (ANOVA) with LSD. Statistical significance was set at *p* < 0.05.

## Results

### Hydrogen alleviated LPS-induced inflammatory infiltration in the lung tissues of mice

**FIGURE 1:**
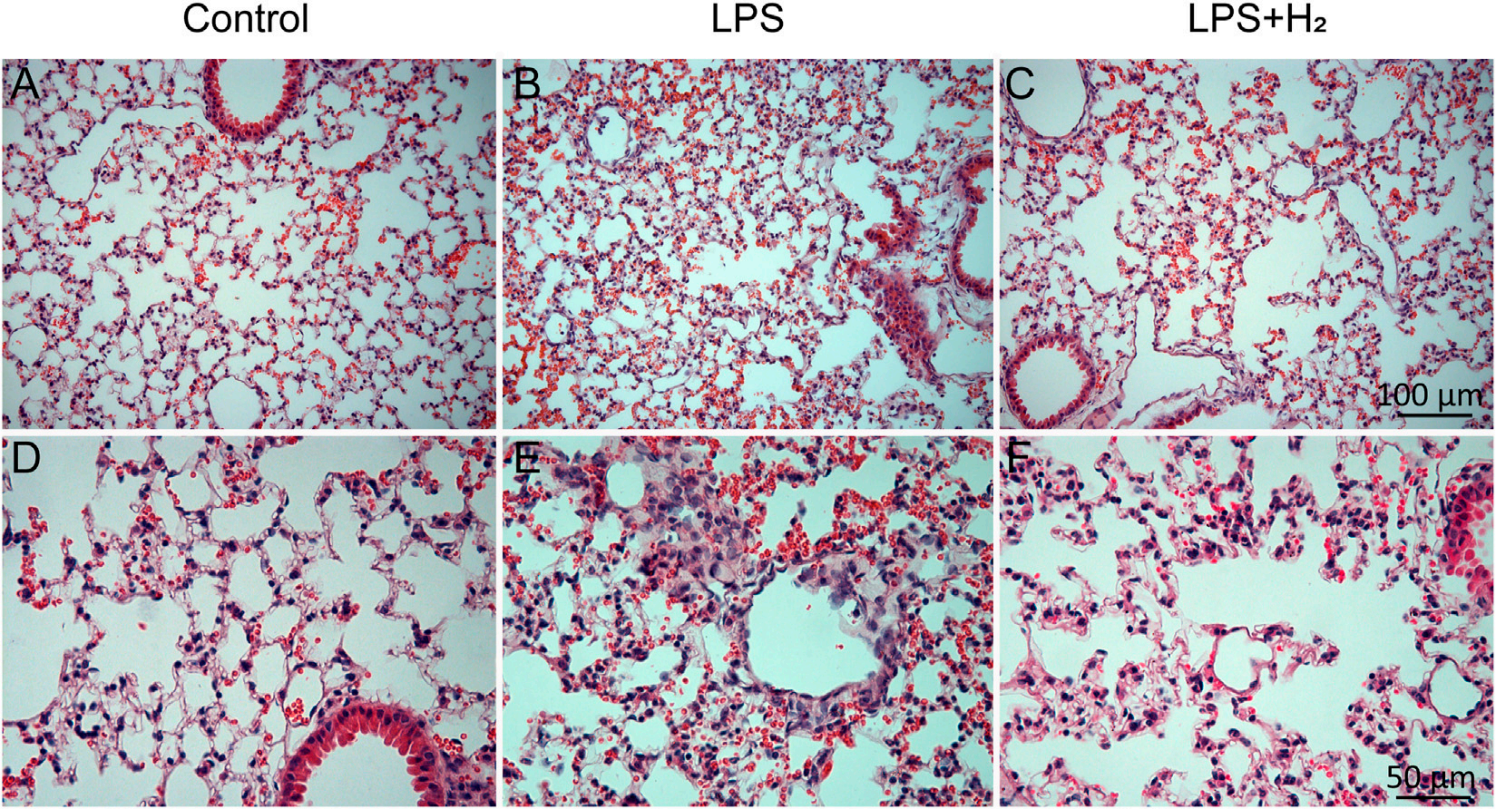
Pathological changes in mouse lung tissue. Three hours after the LPS (2 mg/kg) treatment, obvious inflammatory exudation and leukocyte adhesion and aggregation were observed in the lung tissues of mice **(B,E)**. Hydrogen (42%) administration for 3 h alleviated the pathological inflammatory changes in the tissues **(C,F)**. **(A–C)** scale bar, 100 μm; **(D–F)** scale bar, 50 μm.

Three hours after the LPS challenge, obvious inflammatory exudation and leukocyte adhesion and aggregation were observed in the lung tissues of mice. Hydrogen administration alleviated the pathological inflammatory changes in the tissues (Figure 1).

Hydrogen distinctly, but discriminately, altered level of peritoneal macrophage-derived serum IL-1α and other proinflammatory cytokines induced by LPS in mice in the initial inflammatory phase.

### Hydrogen distinctly, but discriminately, altered level of peritoneal macrophage-derived serum IL-1α and other proinflammatory cytokines induced by LPS in mice in the initial inflammatory phase

**FIGURE 2:**
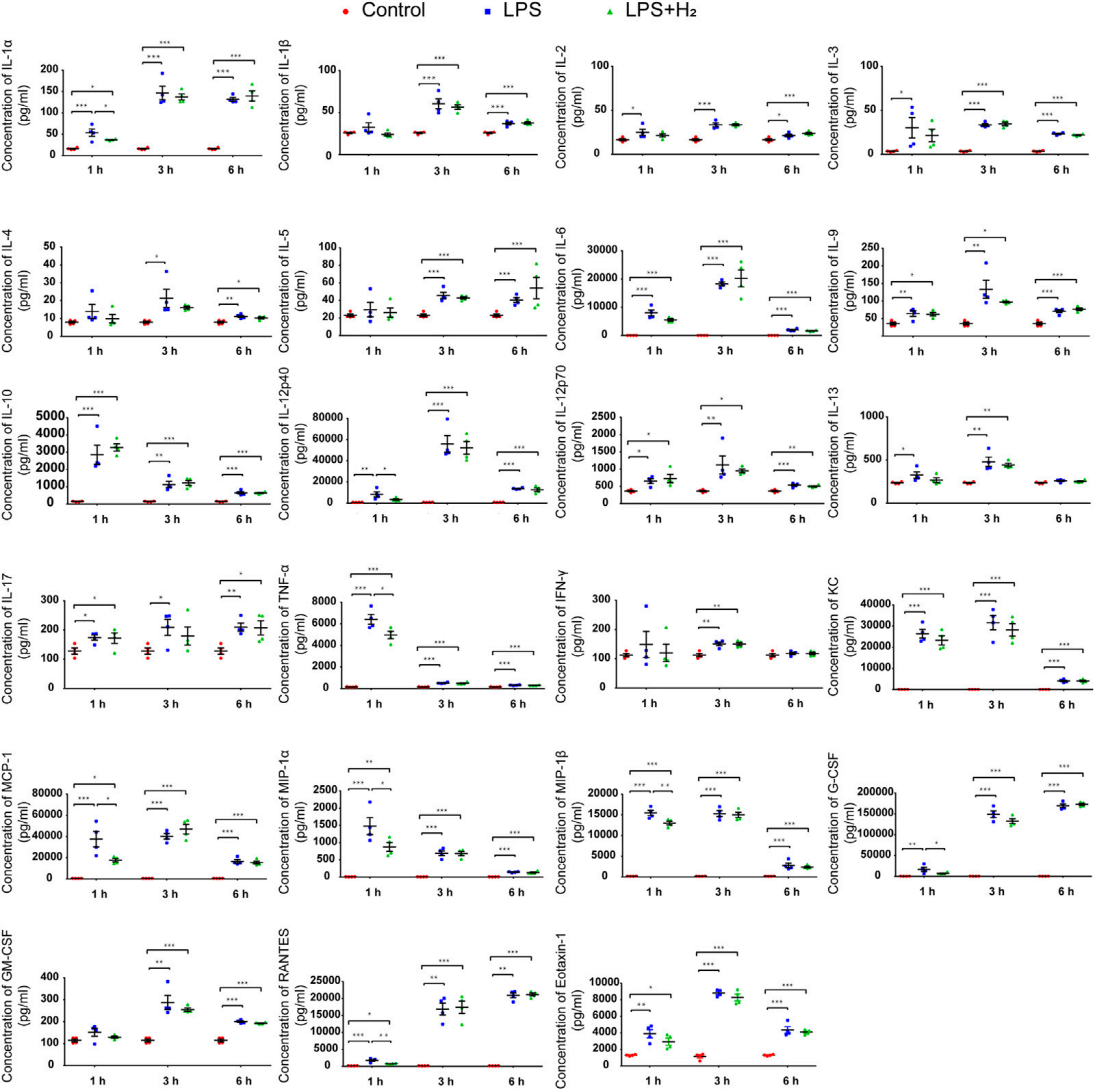
Effects of hydrogen on LPS-induced protein concentrations of 23 cytokines and chemokines in mouse serum. The serum levels of 23 cytokines and chemokines from the mice after LPS (2 mg/kg) and/or 42% hydrogen treatment were analyzed in duplicates at 1, 3, and 6 h time points. Data were expressed as mean ± SEM, *p < 0.05, **p < 0.01, ***p < 0.001, *n* = 4.

After intraperitoneal injection of LPS, an acute systemic inflammatory response was induced immediately, and the concentrations of different cytokines and chemokines in the serum from the mice increased rapidly at different time point from 1 h to 6 h as shown in Figure 2. Hydrogen administration inhibited the secretion of IL-1α, IL-12p40, TNF-α and five chemokines (MCP-1, MIP-1α, MIP-1β, RANTES, and G-CSF) at 1 h; however, it did not significantly alleviate the secretion of other cytokines. Hydrogen had no inhibitory effect on LPS-induced changes in 23 cytokines and chemokines in mouse serum at 3 and 6 h.

**FIGURE 3:**
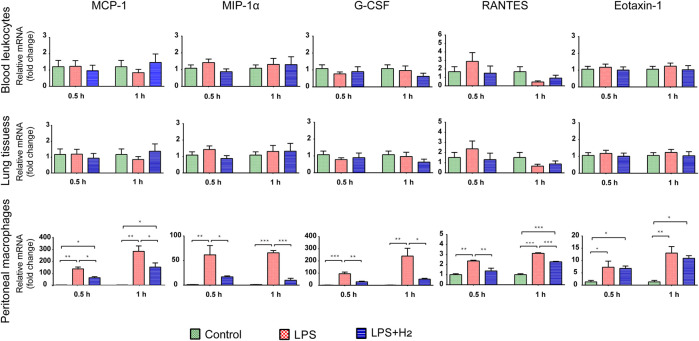
Effects of hydrogen on LPS-induced mRNA expression of five chemokines in mouse lung tissues, leukocytes, and peritoneal macrophages, respectively, and the onset time points. The mRNA levels of MCP-1, MIP-1α, G-CSF, RANTES, and Eotaxin-1 from the mice after LPS (2 mg/kg) and/or 42% hydrogen treatment were analyzed in triplicates at 0.5 h and 1 h time points. Data were expressed as mean ± SEM, *p < 0.05; **p < 0.01; ***p < 0.001; *n* = 3.

To understand the cellular origin of serum proinflammatory cytokines, we analyzed the mRNA expression of MCP-1, MIP-1α, G-CSF, RANTES, and Eotaxin-1 at 0.5, and 1 h in mouse lung tissues, blood leukocytes, and peritoneal macrophages. As shown in Figure 3, the mRNA levels of these chemokines in lung tissues and blood leukocytes showed no significant changes in either control group, LPS group or hydrogen group. In contrast, 0.5 h after LPS injection, the mRNA levels of the five chemokines in peritoneal macrophages immediately and significantly increased and remained elevated to the 1 h time point, with hydrogen exerting obvious antagonistic effect on MCP-1, MIP-1α, G-CSF, RANTES transcription, but not on Eotaxin-1 transcription.

These results indicate that the lung tissues and leukocytes were not the source for chemokine proteins in the blood at an early stage. The chemokines detected in the blood were derived from peritoneal macrophages. LPS rapidly stimulated the transcription of chemokines at an early stage in macrophages in the peritoneal cavity, whereas hydrogen inhibited transcription at an early stage.

### Hydrogen significantly reduced LPS-induced increase in HIF-1α and IL-1α expression.

**FIGURE 4:**
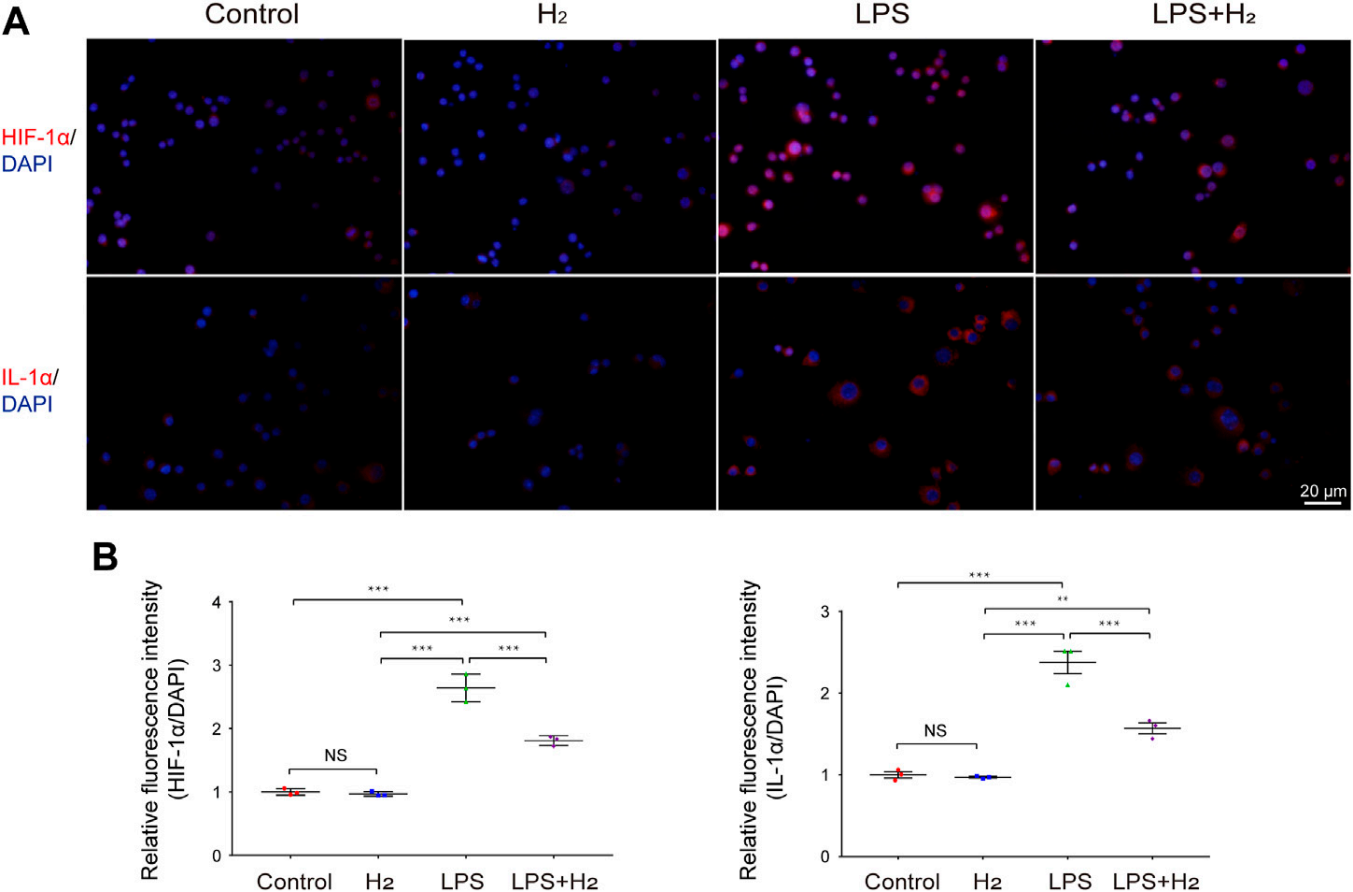
Effects of hydrogen on LPS-induced HIF-1α, and IL-1αexpression in RAW264.7 cells. **(A)** Representative images of HIF-1α and IL-1α activation in RAW264.7 cells in response to 1 μg/mL LPS and/or 60% hydrogen treatments for 0.5 h. Scale bar, 20 μm. **(B)** The HIF-1α, and IL-1α immunofluorescence was quantitated and presented as relative fluorescence intensity (mean ± SEM; **p < 0.01; ***p < 0.001; not significant (NS), *p* > 0.05; *n* = 3).

To understand how hydrogen affects generation of proinflammatory cytokines in macrophages, we analyzed cytoplasmic levels of HIF-1α and IL-1α. The levels of HIF-1α and IL-1α in the cytoplasm of RAW264.7 cells increased rapidly 0.5 h after LPS treatment, whereas this process was significantly reduced after hydrogen administration (Figure 4).

**FIGURE 5:**
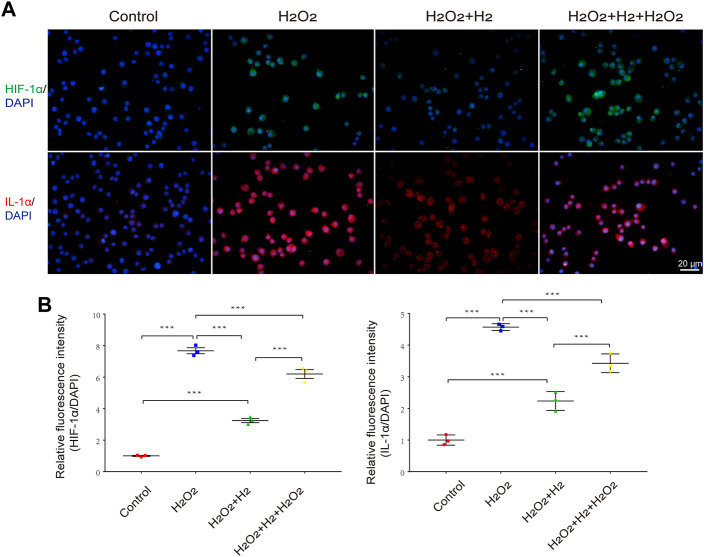
Effects of hydrogen on H_2_O_2_-induced HIF-1α and IL-1α expression in RAW264.7 cells. **(A)** Representative images of HIF-1α and IL-1α activation in response to 31.26 μM H_2_O_2_ and/or 60% hydrogen treatment for 0.5 h in RAW264.7 cells. Scale bar, 20 μm. **(B)** The HIF-1α and IL-1α immunofluorescence was quantitated and presented as relative fluorescence intensity (mean ± SEM; **p < 0.01; ***p < 0.001; *n* = 3).

To provide direct evidence that hydrogen increases HIF-1α and IL-1α levels in macrophages, we administered hydrogen to H_2_O_2_-challenged macrophages *in vitro*. We observed that the expression of HIF-1α and IL-1α in RAW264.7 cells was significantly enhanced by H_2_O_2_ treatment, whereas hydrogen administration significantly blocked this effect. Re-administration of H_2_O_2_ increased the expression of HIF-1α and IL-1α (Figure 5).

## Discussion

LPS, an integral component of the cell wall of Gram-negative bacteria, is an important constituent that causes inflammation in the body (Chen et al., 2017) Because of its small molecular size, it can be quickly absorbed by the rich capillaries in the peritoneal cavity, thus directly activating inflammatory cells to secrete high levels of inflammatory mediators and to trigger a systemic inflammatory cascade reaction (Jardine et al., 2019).

A systemic inflammatory reaction is a complex long-lasting process that includes primary reactions, secondary reactions, chain reactions, amplification effects, marginal effects, compensatory effects, self-regulation, necrosis, and clearance. The later the inflammatory stage, the more complex the serum inflammatory products (Martinez-Garcia et al., 2019). Hydrogen, being reported as valuable treatment but supported with limited information from published studies and evidence from animal experiments, is suggested to work efficiently in combating systemic inflammation (Saramago et al., 2019; Niu et al., 2020). To demonstrate the biological effects of hydrogen inhalation on systemic inflammation, we focused on the period of this study at the initial proinflammatory phase within 12 h (especially within 1–3 h) after the model was created.

In this study, the pathological sections showed that inflammatory cells in small vessels and capillaries began to adhere and gather, and macrophages began to migrate and exude in the lung tissue of mice 3 h after LPS intraperitoneal injection. At 12 h, bilateral diffuse alveolar injury and pulmonary edema were evident and accompanied by a large amount of inflammatory cell exudation, consistent with the findings about LPS-induced pathological alterations of lung (Sun et al., 2021). As the counteraction, lung inflammation was significantly alleviated by hydrogen administration, and both cell infiltration and inflammatory exudation were significantly reduced. Coming into an agreement with this study, Xie et al. (2012) confirmed that hydrogen can inhibit the infiltration of neutrophils and macrophages in lung tissue, Wang et al. (2020) also reported that hydrogen inhalation for 45 min could reduce airway inflammation in patients with asthma and chronic obstructive pulmonary disease (COPD).

In addition, Calvano et al. (2005) focused on blood leukocytes to study the human systemic inflammatory response and reported that the expression of IL-1α, TNF, IL-1β, GRO-α, GRO-β, MCP-1, and IL-8 peaked in the first 2–4 h after LPS administration in normal individuals. For this study, we chose 1, 3, and 6 h time points to collect peripheral blood samples from mice. A 23-Plex protein chip was used to detect changes in seven chemokines and 16 cytokines over a wide range in response to the inflammation reaction and the onset of hydrogen administration. The results showed that hydrogen could inhibit the release of chemokines (MCP-1, MIP-1β, MIP-1α, G-CSF, and RANTES) and IL-1α, IL-12P40, TNF-α to a large extent within 1 h, thus suppressing the exudation of monocytes and granulocytes at the initial stage of inflammation.

Previous researches reported that hydrogen treatment could reduce the levels of TNF-α, IL-1α, IL-1β, IL-6, IL-8, HMGB1, MCP-1, and Egr-1 in lung tissues of animal models (Huang et al., 2010), and pretreatment with hydrogen resulted in cardio-protection during septic cardiomyopathy *via* inhibiting the expression of pro-inflammatory cytokines TNFα, IL-1β, and IL-1 (Tan et al., 2019). Patients with suspected 2019-nCoV had higher plasma levels of IL-2, IL-7, IL-10, G-SCF, IP-10, MCP-1, MIP-1a, and TNF-α (Huang et al., 2020). Recent studies reported that inhalation of a hydrogen/oxygen mixture reduced the severity of dyspnea in patients with COVID-19, and the early use of hydrogen in COVID-19 patients may inhibit the release of cytokines and reduce lung injury (Guan et al., 2020; Yang et al., 2020; Botek et al., 2022; Luo et al., 2022). We previously reported the use of histological and cellular factor analyses to evaluate the effect of hydrogen on ovalbumin-induced sinus mucosal inflammatory reaction (allergic rhinitis, AR) in mice. The results showed that hydrogen inhalation significantly alleviated the symptoms of sneezing and pruritus, reduced the infiltration of inflammatory cells into the mucosa, and decreased the levels of IL-5, IL-13, and MCP-1 in serum, indicating the value of hydrogen in treating allergic diseases (Fang et al., 2018).

Another important problem is to figure out the origin of these chemokines in the blood. To start the investigation, we selected mouse lung tissue cells, blood leukocytes, and peritoneal cavity macrophages to analyze the mRNA expression of five chemokines: MCP-1, MIP-1α, G-CSF, RANTES, and Eotaxin-1. The increasing concentrations of MCP-1, MIP-1α, G-CSF, and RANTES in serum originated from the secretion of macrophages in the peritoneal cavity, with hydrogen significantly suppressed their transcription at an early stage of inflammation (0.5 h).

Macrophages are among the most fundamental immune cell types in the body and are involved in various major diseases (Frodermann and Nahrendorf, 2018; Peterson et al., 2018). In particular, excessive inflammatory activation of macrophages can lead to septicemia (Kumaran Satyanarayanan et al., 2019), which is a key factor in the inflammatory storm generated by CAR T cells in clinical practice (Giavridis et al., 2018) and a major cause of COVID-19 death (Mehta et al., 2020). Clearly, the activation of macrophages is a fundamental factor in the early initiation of an inflammatory response and a key criterion for efficacy of hydrogen in exerting inflammation-suppressing effect. Therefore, where are the key regulatory points where hydrogen inhibits chemokines secreted by macrophages? In this study, we utilized cultured RAW264.7 as a cultured *in vitro* macrophage model and analyzed the changes in HIF-1α, and IL-1α in macrophages under LPS treatment, and also the protective effect of hydrogen. It was observed that LPS sharply increased HIF-1α, and IL-1α concentrations after treatment for 0.5 h. In contrast, hydrogen significantly inhibited this process.

HIF-1α is an important transcription factor for the regulation of the hypoxia response. In recent years, many studies have confirmed that HIF-1α is closely related to the occurrence and development of inflammation and plays a strong pro-inflammatory role in the pathogenesis of rheumatoid arthritis, psoriasis, pulmonary hypertension, and tumors (Kriebel et al., 2018; Luo et al., 2019; Zhang et al., 2020; Zhu et al., 2020). In such manner, studies were conducted in which IL-1α was shown to be regulated by HIF-1α, and a change in HIF-1α expression altered the tumor-promoting effect of IL-1α (Xuan and Wang, 2017). Heun et al. also confirmed that HIF-1α can induce IL-1α production in inflammatory cells at an early stage of wound healing in a non-hypoxic manner (Heun et al., 2017).

In experimental designs, LPS is commonly used to induce HIF-1α mRNA accumulation and HIF-1α protein synthesis in macrophages in a ROS-dependent manner (Nishi et al., 2008), promote HIF-1α binding to hypoxia response elements (HREs) in target genes, activates transcription of hundreds of downstream target genes, and enhances the inflammatory response. Inflammation, in turn, aggravates hypoxia in the microenvironment, leading to further upregulation of HIF-1α and promoting its nuclear aggregation (Golz et al., 2015). This vicious cycle may be an important mechanism in the cascade of inflammatory factors, indicating that HIF-1α is a key node in this cycle. Therefore, regulation of HIF-1α is an important drug target for controlling and alleviating the occurrence and development of inflammation (Heun et al., 2017; Xuan and Wang, 2017; Chowdhury et al., 2018).

On the other hand, IL-1α is an important member of the IL-1 family. In its stable state, it is localized in the nucleus; however, after activation, its expression is rapidly upregulated, and it is released as an early warning signal after dissociation from the nucleus to initiate the inflammatory process (Kany et al., 2019). IL-1α is widely expressed *in vivo*, and its precursor and mature forms are bioactive. Due to its various characteristics, it has been recognized as the “inflammatory apical activator” (Malik and Kanneganti, 2018) and is the source of initiation of the inflammatory response (Malik and Kanneganti, 2018). In our experiment after LPS challenge, the level of IL-1α in blood increased rapidly in the first hour, reached a peak at the third hour, and continued until the sixth hour, but the process was neutralized by hydrogen which inhibited IL-1α, capturing the key regulator of inflammatory damage and inhibiting its occurrence at source.

In addition to our research, studies have shown that IL-1α secretion is precisely regulated by HIF-1α (Singh et al., 2019), with 2MeOE2 as HIF-1α inhibitor that stop the activation and transcriptional translation of IL-1α, preventing its release and activation from the nucleus, thereby preventing the initiation and development of inflammatory procedures. Thus, IL-1α has become an important drug target in many diseases (Dinarello et al., 2012) such as sepsis (Benjamin et al., 2018), chronic toxoplasma infection (Batista et al., 2020), inflammatory bowel disease, Crohn’s disease, and diabetes mellitus (Collier et al., 2021).

Cytokines play a key role in homeostasis and host defense, and a small change in cytokine levels may have a large effect. Therefore, potential risks exist in simply inhibiting their secretion, with many of these drugs having serious side effects. However, hydrogen inhalation has emerged as a promising protocol for the treatment of various inflammation-relevant diseases owing to its safety, convenience, and efficacy. In addition, its low molecular weight allows it to easily cross cell membranes and spread into organelles such as the nucleus and mitochondria where it can have rapid effects. Our findings provide direct experimental evidence for safe and effective application of hydrogen-assisted treatment to inhibit inflammation.

## Conclusion

Hydrogen has the potential to inhibit inflammation by inhibiting HIF-1α and IL-1α release at early time points. The target of the anti-LPS-induced inflammatory action of hydrogen is chemokines in macrophages in the peritoneal cavity. This study provides direct experimental evidence for translational application of a hydrogen-assisted protocol to quickly control inflammation.

## Data Availability

The raw data supporting the conclusion of this article will be made available by the authors, without undue reservation.
