# Supplementary material for: Potent anti-inflammatory responses: Role of hydrogen in IL-1α dominated early phase systemic inflammation
Source: Front Pharmacol. 2023 Mar 17;14:1138762. doi: 10.3389/fphar.2023.1138762 (PMC10063881; doi:10.3389/fphar.2023.1138762)
Supplement: Supplementary file 1 [file Presentation1.pdf]

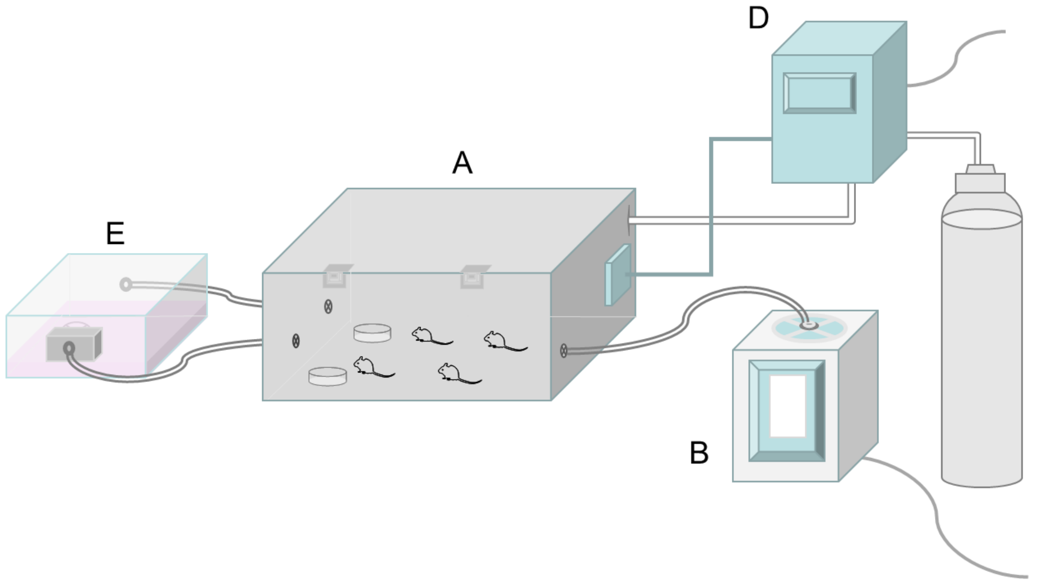

Fig. S1. Schematic diagram of the hydrogen treatment warehouse and hydrogen supply device. **(**A) Hydrogen treatment warehouse. (B) Hydrogen and oxygen generator. (C) Nitrogen gas source. (D) Oxygen controller (E) Flow filter chamber.
